# With great power comes great risk: High ureteral stricture rate after high-power, high-frequency Thulium fiber laser lithotripsy in ureteroscopy

**DOI:** 10.1007/s00345-025-05553-0

**Published:** 2025-04-18

**Authors:** Riccardo Villani, Thibaut Dominique Liernur, Olivier Laurent Windisch, Massimo Valerio, Fabian Thierry Schoofs, Jia-Lun Kwok, Alba Sierra, Daniel Eberli, Christophe Iselin, Olivier Traxer, Etienne Xavier Keller

**Affiliations:** 1 Department of Urology, University Hospital Zurich, University of Zurich, Zurich, Switzerland; 2 Department of Surgery, Service of Urology, University Hospital Geneva, University of Geneva, Geneva, Switzerland; 3 Department of Urology, Tan Tock Seng Hospital, Singapore, Singapore; 4 Progressive Endourological Association for Research and Leading Solutions (PEARLS), Paris, France; 5 Section of Endourology, European Association of Urology, Arnhem, The Netherlands; 6 Urology Department, Hospital Clinic de Barcelona, Villarroel 170, Barcelona, 08036 Spain; 7 Young Academic Urologists (YAU), Endourology & Urolithiasis Working Group, Arnhem, The Netherlands; 8 Cabinet privé, 76b Av. de la Roseraie, Geneva, Switzerland; 9 Service d’Urologie, Sorbonne Université, Hôpital Tenon, Paris, France; 10 Sorbonne Université, GRC n°20, Groupe de Recherche Clinique sur la Lithiase Urinaire, Hôpital Tenon, Paris, F-75020 France

**Keywords:** Ureteroscopy, Lithotripsy, Thulium fiber laser, Ureteral stricture, Laser presets, Power

## Abstract

**Purpose:**

To compare the safety and efficacy of Thulium Fiber Laser (TFL) using either manufacturer presets (MP) or individualized presets (IP) in ureteroscopy.

**Methods:**

Multi-institutional, retrospective analysis on the first patients treated with SOLTIVE^®^ Premium (Olympus Medical Systems^®^) TFL in Switzerland in 2020. MP were used at the University Hospital of Geneva, while IP were used at the University Hospital of Zurich. Patient demographics, stone characteristics, and procedural details were collected. Primary outcome was postoperative ureteral stricture (US). Secondary outcome was stone-free rate (SFR).

**Results:**

A total of 158 patients were analyzed, 79 in each group. Demographics were similar between the two groups, except for a lower pre-stenting rate in the MP group (56% vs. 91%; *p* < 0.001) and a higher rate of ureteral access sheath use in the MP group (65% vs. 44%; *p* = 0.011). No significant differences in stone burden (median stone diameter 9 mm, median stone volume 267 mm^3^), nor in the rate of impacted ureteral stones (29% vs. 34%; *p* = 0.49). Mean power, maximal power, frequency settings, and energy consumption were significantly higher in the MP group. US rate was 11% in MP group compared to 1% in IP group (*p* = 0.009). MP were a significant predictor of US on multivariable analysis (OR 12.4; *p* = 0.02), independently from impacted ureteral stones. No difference in SFR between groups (85% and 84%; *p* = 0.67).

**Conclusion:**

High-power, high-frequency laser settings from manufacturer laser presets increase the risk of US, without improving SFR. Future studies shall further evaluate optimal laser settings depending on patient characteristics and intraoperative situation.

**Supplementary Information:**

The online version contains supplementary material available at 10.1007/s00345-025-05553-0.

## Introduction

Ureteroscopy (URS) is currently the most widely used operative technique for the treatment of urolithiasis [1]. For ureteroscopic laser lithotripsy, a series of pulsed lasers can be used: the Holmium: YAG laser, the pulsed Thulium: YAG laser and the superpulsed Thulium Fiber Laser (TFL) [2–4]. The TFL has shown superiority over the Holmium: YAG in several aspects (e.g. higher stone ablation, smaller stone fragments, lower retropulsion, smaller laser fibers, higher stone-free rate (SFR), shorter operative time), but safety warnings and reports from urologists surfaced the concern if TFL is associated with a higher risk of ureteral stricture (US) [2, 5–7]. The proposed mechanisms are wide and not sustained by robust evidence, with conflicting findings between in vitro studies [7–11]. The question, then, is how far we can push laser settings during URS to maximize efficacy while ensuring the patient’s safety. An analysis of a Twitter pool of experts from October 2021 resulted in a high degree of variability in preferred laser settings, with no consensus [12]. De facto, many urologists worldwide rely on laser presets provided by manufacturers, which show a high degree of variability too [13].

**Fig. 1:**
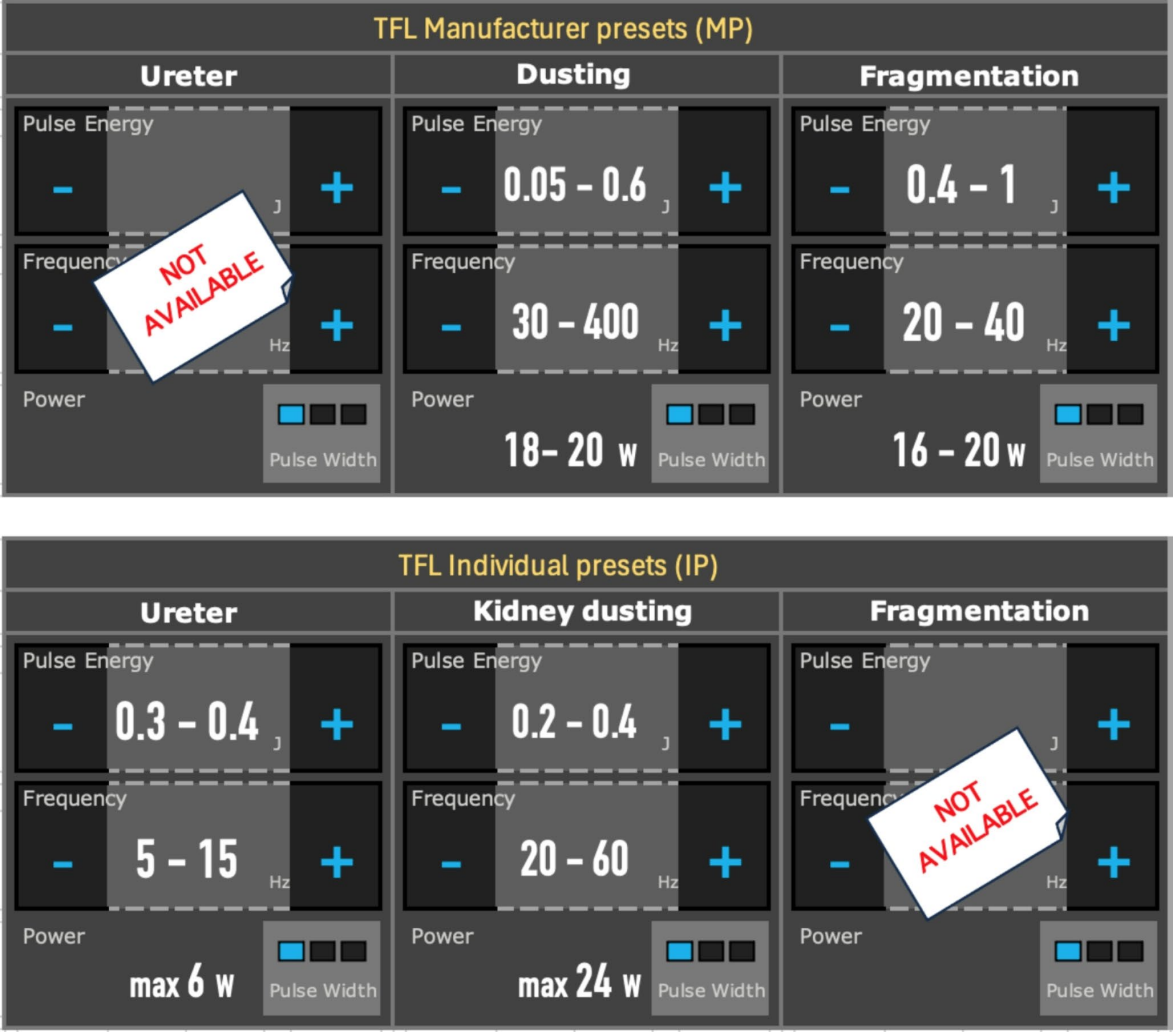
Graphical user interface (GUI) comparison of manufacturer presets (MP) and individual presets (IP) for the Olympus SOLTIVE^®^ Premium Thulium Fiber Laser (TFL) in 2020. Regarding manufacturer presets: Until July 2021, no manufacturer presets specifically intended for the ureter were available, nor was there any indication that the existing presets were designed for use in the kidney, but not the ureter. Regarding individual presets: Since the TFL was primarily introduced as a “dusting” laser, there were no individual presets dedicated to stone “fragmentation”

In this context, the first two hospitals using TFL in clinical routine in Switzerland followed different approaches for laser presets: the University Hospital of Geneva (Hôpitaux Universitaires de Genève, HUG) used manufacturer presets (MP), while the University Hospital Zurich (Universitätsspital Zürich, USZ) used individual presets (IP) based on recommendations from a fellowship-trained urologist who had established internal hospital guidelines on laser settings *(*Fig. 1*)* [14–16].

This difference in practice was the basis for a comparison between patients treated at the HUG and USZ for analysis of safety and efficacy of TFL laser lithotripsy.

## Materials and methods

We conducted a multi-institutional and retrospective analysis of the first patients who underwent URS-lithotripsy with the SOLTIVE^®^ Premium TFL (Olympus Medical Systems^®^, Tokyo, Japan) in Switzerland, namely at the HUG and USZ. The HUG discontinued the use of the TFL after 79 patients. Accordingly, the first 79 patients treated at the USZ were included for analysis. Patients with history of prior reconstructive ureteral surgery or pre-existing strictures were excluded, as well as patient who underwent URS with stone extraction, but no laser lithotripsy. Data was extracted from the hospital’s medical information system. The following patient characteristics were collected: age, sex, BMI, diabetes mellitus history, ASA score, and prior history of ipsilateral URS. Data regarding characteristics and location of the stones were also collected, including their location within the kidney or ureter (proximal, mid and distal ureter), number of stones, maximal diameter of the largest stone, stone volume, maximal stone density measured in Hounsfield Units (HU), as well as presence of hydronephrosis at the time of diagnosis. To accurately estimate the stone volume, we employed the ellipsoid volume formula (4/3 π a*b*c, where a, b, and c represent the radius of the stone in three dimensions, calculated from pre-operative Computer-Tomography (CT) scans in both axial and coronal views). In case of multiple stones, we summed up each stone volume [17]. The presence of an impacted ureteral stone was retrieved from the medical files. The general practice in both institutions was to consider a ureteral stone as impacted if at least one of the following criteria was met: (a) presence of ureteral dilation proximal to the stone and moderate to severe hydronephrosis (grade ≥ 2) [18]; (b) difficulty passing a guidewire or ureteral catheter beyond the ureteral stone at first attempt; (c) stone remaining in the same location on two separate imaging studies taken at least two months apart [19]. Regarding the surgical procedure, we collected data on unilateral vs. bilateral URS, pre- and postoperative Double-J (DJ) ureteral stenting (pre-stenting in both institutions usually for 10–20 days; post-operative DJ-stenting usually 5–14 days), and ureteral access sheath (UAS) use. *Detailed surgical procedure steps and instruments are provided in Supplementary Material*. Since all surgeries were performed in a university hospital setting, each patient was treated by either a urologist with > 100 URS experience or by a urology resident under the supervision of an experienced urologist.

Laser settings were retrospectively extracted from the SOLTIVE^®^ Premium laser generators, which have a record of the following parameters for each treated patient: type of laser fiber, active lasing time, total delivered energy, maximal power setting, as well as lowest and highest frequency settings. Mean power was calculated as total energy divided by lasing time. Mean laser energy consumption was calculated as total energy divided by stone volume (J/mm^3^) [20].

Primary outcome was the presence of postoperative ureteral stricture (US). US was defined, according to Moretto et al., as signs of urinary flow impairment due to ureteral narrowing, associated with complications (such as pain, recurrent infections, or loss of renal function) and/or leading to re-intervention [21, 22]. In both institutions, patients underwent clinical evaluation and kidney-ultrasonography six weeks postoperatively. According to institutional guidelines, patients showing symptoms, impaired renal function, or hydronephrosis suggesting US were thoroughly evaluated. In such cases, the option of a retrograde ureteropyelography with diagnostic URS and/or dynamic scintigraphy was discussed. Other safety outcomes included intra-operative complications, as well as early (< 1 month) and late (≥ 1 month) postoperative complications, classified according to the Clavien-Dindo grading system [23, 24].

Secondary outcome was the SFR, defined as the endoscopic absence of fragments greater than 2 mm and absence of fragments greater than 2 mm in follow-up imaging (ultrasonography in all patients, with additional imaging (e.g. CT) based on the surgeon’s discretion) [25].

### Statistical analysis

Associations between categorical variables were analyzed with Chi Square tests or Fischer’s exact tests, as appropriate. Differences between continuous variables were analyzed with independent t-tests or Mann-Whitney U tests, as appropriate. Correlations between continuous variables was analyzed with Pearson correlations tests or Spearman’s rho test, as appropriate. Logistic regression was used for the analysis of predictors of dichotomic outcomes. Patients with bilateral URS were included in a per-patient analysis. All statistical tests were two-sided and *p*-values < 0.05 were considered significant. Statistical analysis and graph plotting were performed with IBM SPSS Statistics 29.0 (IBM Corp., Armond, NY, USA).

## Results

A total of 158 patients were available for analysis, 79 patients in each group, all treated between June 2020 and May 2021. Mean age was 53 years.

All patients underwent preoperative CT scan to assess stone burden. The majority of procedures were performed for kidney stones rather than ureteral stones. Specifically, 70% of patients had kidney stones (with/without concomitant ureteral stones), while 53% had ureteral stones (with/without concomitant kidney stones). There was a significant correlation between maximal stone size and stone volume (Spearman’s rho 0.92; *p* < 0.001; Supplementary Fig. 1). Table 1 provides comprehensive patient characteristics.

**Table 1:** Patient characteristics

Baseline characteristics					Procedural characteristics				
Variable	**Overall (** ***n*** ** = 168)**	**Manufacturer laser presets (** ***n*** ** = 79)**	**Individual laser presets (** ***n*** ** = 79)**	p-value	**Variable**	**Overall (** ***n*** ** = 168)**	**Manufacturer laser presets (** ***n*** ** = 79)**	**Individual laser presets (** ***n*** ** = 79)**	p-value
Age (mean, SD)	53 (17)	52 (19)	54 (15)	0.48*	**UAS use**	86 (54%)	51 (65%)	35 (44%)	0.011**
Male : female ratio	2.1: 1	2.0: 1	2.2: 1	0.87**	**Postoperative DJ-stenting**	156 (99%)	79 (100%)	77 (98%)	0.16**
BMI (mean, SD) (kg/m^2^)	26 (5)	26 (5)	26 (5)	0.76*	**Laser fiber core diameter**				< 0.001**
					**150 μm**	84 (53%)	5 (6%)	79 (100%)	
					**200 μm**	68 (43%)	68 (86%)	0 (0%)	
					**365 μm**	6 (4%)	6 (8%)	0 (0%)	
Diabetes mellitus	23 (15%)	7 (9%)	16 (20%)	0.04**	**Active lasing time (mm: ss) (median**, **IQR)**	06:28 (01:56–16:36)	04:38 (01:37–09:37)	13:05 (02:42–23:33)	< 0.001***
ASA score				0.65**	**Total energy (KJ)**	6.26 (1.63–17.1)	6.50 (2.28–16.38)	5.98 (9.03–17.50)	0.41***
1	17 (11%)	9 (11%)	8 (10%)						
2	94 (59%)	50 (63%)	44 (56%)						
3	41 (26%)	17 (22%)	24 (30%)						
4	6 (4%)	3 (4%)	3 (4%)						
Prior history of URS	44 (28%)	22 (28%)	22 (28%)	1.00**	**Energy consumption (J/mm**^**3**^**) (median**, **IQR)**	18 (7–32)	20 (11–44)	17 (7–26)	0.031***
Stone location				0.94**	**Mean power (W) (median**, **IQR)**	19 (8–20)	20 (20–25)	8 (6–15)	< 0.001***
Kidney	67 (43%)	33 (42%)	34 (43%)						
Ureter	48 (30%)	25 (32%)	23 (29%)						
Both	43 (27%)	21 (26%)	22 (28%)						
Ureteral stones’ location				0.18**	**Maximal power setting (W) (mean**, **SD)**	21 (13)	27 (13)	15 (9)	< 0.001*
Proximal	54 (59%)	54 (59%)	31 (69%)						
Mid	25 (28%)	25 (28%)	8 (18%)						
Distal	9 (10%)	9 (10%)	4 (9%)						
Multiple	3 (3%)	3 (3%)	2 (4%)						
Number of stones (median, IQR)	1 (1–3)	1 (1–3)	2 (1–3)	0.28***	**Lowest frequency setting (Hz) (median**, **IQR)**	40 (10–200)	200 (100–200)	14 (5–30)	< 0.001***
Maximal stone size (mm) (median, IQR)	9 (7–13)	9 (7–13)	9 (7–13)	0.39***	**Highest frequency setting (Hz) (median**, **IQR)**	100 (40–400)	400 (200–400)	40 (30–100)	< 0.001***
Stone volume (mm^3^) (median, IQR)	267 (110–751)	269 (117–565)	264 (110–857)	0.67***	**Postoperative outcomes**				
Maximal stone density (HU) (mean, SD)	1142 (391)	1108 (382)	1177 (399)	0.27*	**Variable**	**Overall (** ***n*** ** = 168)**	**Manufacturer laser presets (** ***n*** ** = 79)**	**Individual laser presets (** ***n*** ** = 79)**	p-value
Impacted stone	50 (32%)	23 (29%)	27 (34%)	0.49**	**Intraoperative complications**	Total: 8 (5%)	Total: 3 (4%)	Total: 2 (3%)	0.15**
					**Ureteral perforation**	6 (4%)	2 (3%)	1 (1%)	
					**Renal pelvis perforation**	1 (1%)	1 (1%)	0 (0%)	
					**Priapism**	1 (1%)	0 (0%)	1 (1%)	
Preoperative hydronephrosis ≥ Grade 2	47 (30%)	21 (27%)	26 (33%)	0.38**	**Early postoperative complications**	Total: 8 (5%)			
	Total: 9 (11%)	Total: 5 (6%)	0.47**						
					**CD I**				
					**Hematuria**	2 (1%)		0 (0%)	
					**CD II**		2 (3%)		
					**UTI**	4 (3%)		3 (4%)	
					**Delirium**	1 (1%)	1 (1%)	1 (1%)	
					**CD IV**		0 (0%)		
					**TIA**	1 (1%)	0 (0%)	1 (1%)	
Bilateral URS	9 (6%)	4 (5%)	5 (6%)	0.73**	**Late postoperative complications**	Total: 10 (6%)	Total: 9 (11%)	Total: 1 (1%)	0.009**
					**CD IIIb**				
					**Ureteral stricture**	10 (6%)	9 (11%)	1 (1%)	
					**Others**	0 (0%)	0 (0%)	0 (0%)	
Pre-stenting	116 (73%)	44 (56%)	72 (91%)	< 0.001**	**Stone free status**	132 (84%)	65 (82%)	67 (85%)	0.67**

*T-test, **Chi-square, ***Mann-Whitney U Test

SD = standard deviation; IQR = interquartile range; BMI = body mass index; ASA = American Society of Anesthesiologists; URS = ureteroscopy; HU = Hounsfield units; UAS = ureteral access sheath; TIA = transient ischemic attack; CD = Clavien-Dindo

Among baseline patient characteristics, diabetes mellitus was less prevalent in the MP group compared to the IP group (9% vs. 20%; *p* = 0.04). Additionally, the MP group had a significantly lower proportion of pre-stented cases (56% vs. 91%; *p* < 0.001). No other baseline variables showed significant differences between the two groups. Specifically, stone burden was comparable (median maximal stone size: 9 mm in both groups; *p* = 0.39; median stone volume 269 mm³ in the MP group and 264 mm³ in the IP group; *p* = 0.67). Similarly, the rate of impacted ureteral stones did not differ significantly between the MP and IP group (29% vs. 34%, respectively; *p* = 0.49).

**Fig. 2:**
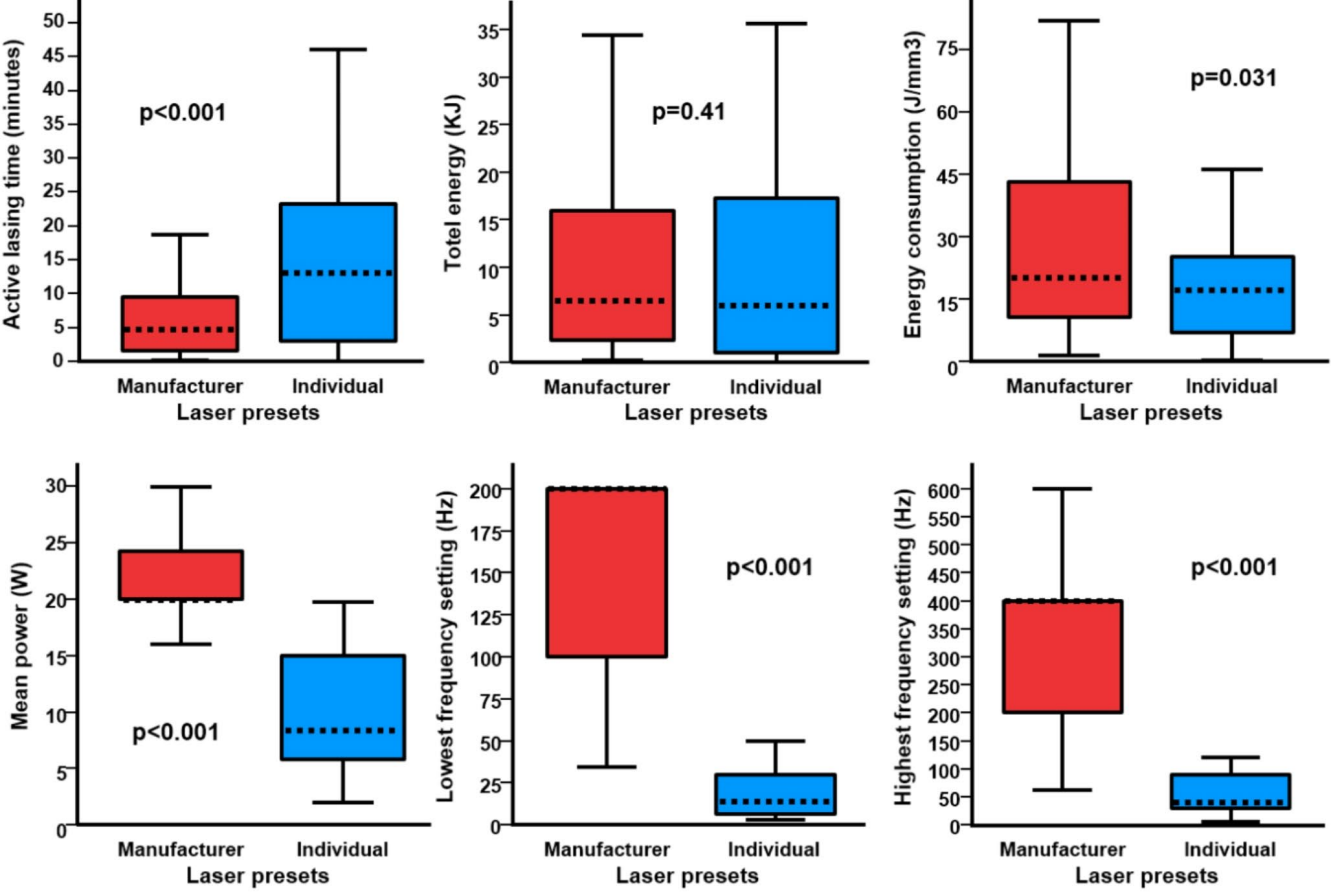
Distribution of laser settings retrieved from the laser generators. Boxplots with median as dotted lines, boxes as 25th to 75th percentiles, and whiskers as 10th to 90th percentiles

Significant differences in procedural characteristics were observed between the two groups. The MP group demonstrated higher rate of UAS use, shorter active lasing times, higher energy consumption (J/mm³), higher mean power, higher maximal power settings, and higher laser frequency settings (applicable to both lowest and highest frequency settings) compared to the IP group *(*Table 1*and* Fig. 2*).*

No patient was lost to follow-up, and all patients underwent at least one first postoperative control within 3 months after surgery. Nine cases of US occurred in the MP group (11%) compared to one case (1%) in the IP group (*p* = 0.009). Impacted stone status, absence of pre-stenting, manufacturer laser presets, increasing total laser energy as well as increasing mean power were found as predictors of US on univariable analysis *(*Table 2*)*. On multivariable analysis, two independent variables emerged as predictors of US: impacted stone status (OR 7.10 (CI 1,67–30,1); *p* = 0.008) and manufacturer laser presets (OR 12,4 (CI 1,48–104); *p* = 0.02) *(*Table 2*)*.

**Table 2:** Logistic regression analysis for predictors of ureteral stricture

Variable	Univariable		Multivariable*	
	**OR (95% CI)**	***p***	**OR (95% CI)**	***p***
Age (continuous)	0.98 (0.94–1.02)	0.32		
Male vs. female	4.59 (0.57–37.3)	0.15		
BMI (kg/m2) (continuous)	1.03 (0.91–1.17)	0.64		
Diabetes mellitus	1.4 (0.3–7.6)	0.62		
Maximal stone size (mm) (continuous)	1.04 (0.94–1.15)	0.44		
Stone volume (mm^3^) (continuous)	1.00 (1.00–1.00)	0.92		
Impacted ureteral stone	5.70 (1.41–23.1)	**0.015**	7.10 (1.67–30.1)	**0.008**
Preoperative hydronephrosis ≥ Grade 2	1.63 (0.44–6.06)	0.47		
Failed primary URS attempt	1.6 (0.3–8.1)	0.57		
Pre-stenting	0.2 (0.06–0.8)	**0.022**		
UAS use	0.8 (0.2–3.0)	0.77		
Manufacturer (MP) vs. individual laser presets (IP)	10.0 (1.24–81.2)	**0.031**	12.4 (1.48–104)	**0.02**
Laser fiber core diameter				
150 μm	1.00 (Ref.)			
200 μm	4.71 (0.94–23.5)	0.059		
365 μm	8.20 (0.63–106.6)	0.11		
Active lasing time (s) (continuous)	1.00 (1.00–1.00)	0.64		
Total energy (KJ) (continuous)	1.02 (1.00–1.04)	**0.026**		
Laser energy consumption (J/mm^3^)	1.00 (0.99–1.02)	0.60		
Mean power (W)	1.03 (1.00–1.06)	**0.046**		
Maximal power setting (W)	1.02 (0.98–1.07)	0.28		
Lowest frequency setting (Hz)	1.00 (0.99–1.01)	0.12		
Highest frequency setting (Hz)	1.00 (0.99–1.01)	0.16		
Intraoperative ureteral or pelvic perforation	3.18 (0.34–30.2)	0.31		

*Only variables retained in a forward conditional multivariable analysis with an entry level at ≤ 0.05 and removal cut-off at ≥ 0.1 were retained in the model

OR = odds ratio; 95% CI = 95% confidence interval; BMI = body mass index

Detailed patient characteristics and management of the 10 patients with US are presented in *Supplementary Table 1*. All patients had ureteral stones at initial diagnosis. All patients underwent retrograde ureteropyelography to locate and measure the US. The location of the US corresponded to the original stone location in 4 from 10 patients (40%), while further distally to the stone location in 4 patients (40%) and more proximally in the remaining 2 patients (20%). Most cases were managed with laparoscopic DaVinci-assisted ureteroplasty (using buccal mucosal graft or appendix flap) or ureteral reimplantation (50%) or balloon dilation and ureteral stenting (33%). Additionally, one case required nephrectomy, while another resolved spontaneously. Patients who underwent reconstruction were followed-up with clinical, ultrasound and renal function assessments for a minimum of 2 years with no loss of follow-up, showing excellent outcomes after reconstructive surgery, with no evidence of recurrence.

As for intraoperative, early postoperative, and late postoperative complications, no significant differences were observed between the MP and IP groups.

SFR did not significantly differ between groups (85% and 84% in the MP and IP group; *p* = 0.67).

## Discussion

To the best of our knowledge, this is the first clinical study confirming the risks associated with the use of high-power, high-frequency settings from manufacturer laser presets in the context of ureteroscopic lithotripsy. Specifically, manufacturer laser presets led to a significantly higher risk of postoperative US (OR = 12.4; *p* = 0.02 on multivariable analysis), compared to more conservative, individualized laser presets, with no difference in SFR between both groups. Most cases of US required major surgical intervention. What had been previously hypothesized in several in vitro studies [7, 26] and highlighted by safety complaints [27–33], is now supported by compelling evidence provided by this study. It serves as a crucial caveat to all urologists performing ureteroscopic laser lithotripsy, underscoring the need for careful evaluation and adjustment of laser settings.

Recent systematic reviews have reported an overall 3% rate of US after URS [22, 23]. In the present study, the US rate in the MP group was 11%, arguably exceeding the safety threshold for this procedure. Understandably, the hospital involved (HUG) opted to discontinue the use of the TFL within less than a year after its acquisition. In contrast, use of IP resulted in a 1% US rate, indicating that ureteroscopic laser lithotripsy can be performed safely with the TFL when using IP.

Two critical factors likely explain the association between laser settings and US formation: high-power and high-frequency. High-power can result in excessive and rapid local energy delivery, potentially causing thermal tissue damage, which may ultimately lead to US formation [34–36]. Similarly, high-frequency is associated with suboptimal energy delivery to the stone surface, increasing the likelihood of inadvertent targeting of ureteral wall rather than the ureteral stone [26, 37].

Ultimately, high-power is mostly associated with high-frequency, and vice versa, because of the relatively narrow range of pulse energy used for laser lithotripsy (predominantly 0.4–1.5 J) [38]. In the present study, mean power, maximal power, lowest and highest frequency settings were all significantly higher in the MP group, compared to the IP group. Additionally, mean power was revealed as a significant predictor of US on univariable analysis (OR = 1.03 for each additional increase of 1 W; *p* = 0.046). Finally, laser energy consumption was significantly higher in the MP group compared to the IP group: 20 J/mm^3^ vs. 17 J/mm^3^ (*p* = 0.031) (the lower, the better, as highlighted by recent reports) [39, 40]. This difference in laser energy consumption is likely attributed to less precise targeting of the stone surface with high-frequency settings. This hypothesis is supported by the findings of two in vitro studies where higher frequency settings were associated with a less effective delivery of laser energy to the stone surface [41, 42].

Poor stone targeting due to impaired visibility during stone dust production and delayed human reaction time when off-target further exacerbates the hazards associated with high-frequency lithotripsy [38]. In our study, most patients with US had laser frequency settings ranging from 100 to 400 Hz. Given a mean visual reaction time of at least 250 ms [43], most surgeons would likely release the laser pedal only after delivering 25 to 100 laser pulses in case of poor stone targeting or reduced visibility. These excessive, uncontrolled laser pulses not only possibly account for the observed differences in laser energy consumption, but might also have contributed to the formation of US in this study.

Presence of an impacted ureteral stone emerged as the second significant and independent predictor of US on multivariable analysis (OR = 7.10; *p* = 0.008). This finding aligns with a recent systematic review and meta-analysis, which confirmed impacted stone as an established predictor of US (OR = 7.47; *p* = 0.01, based on 5 studies including 13550 patients) [22]. In the present cohort, seven out of ten patients who developed US (70%) had impacted stones. However, 43 other patients with impacted stones did not develop US, underscoring the influence of competing risk factors such as high-power, high-frequency laser lithotripsy.

All cases of US occurred in patients with stones located in the ureter at initial diagnosis. Noteworthy, strictures occurred at the same location as the original stone in only 40% of all cases with US. This observation suggests that either stones were displaced between initial diagnosis and ureteroscopic laser lithotripsy, or that thermal tissue damages may have occurred distantly from the site of local laser energy delivery. Consequently, and until proven otherwise, high-power high-frequency laser lithotripsy should be considered hazardous for both ureteral and kidney stones. Future studies shall verify the hypothesis of distant heat-generated tissue damages by protocolling the site of laser emission in a prospective manner.

Interestingly, pre-stenting significantly reduced the risk for US on univariable analysis (OR = 0.2; *p* = 0.022). Although this finding may partly reflect practice differences between the two hospitals (56% pre-stenting in the MP group vs. 91% in the IP group; *p* < 0.001), it is plausible to hypothesize that pre-stenting contributes to US prevention by dilating the ureter and improving irrigation flow, therefore minimizing risks of ureteral trauma and optimizing conditions of laser lithotripsy [44]. Overall, pre-stenting is associated with higher SFR and fewer intra- and postoperative complications [45]. Further studies are needed to explore the impact of pre-stenting on reducing the risk of US.

Regarding the use of UAS, it was more frequently employed in the MP group (65% vs. 44% in the IP group; *p* = 0.011). Nevertheless, UAS use was not identified as a predictor of US. These findings are consistent with previous studies, which suggest that there is no established association between UAS placement during URS and the development of US [19, 46].

Neither stone size (maximal diameter) nor stone volume emerged as predictors of US in our study. Conversely, total delivered energy was identified as significant predictor of US on univariable analysis (OR = 1.02 for each additional 1 kJ; *p* = 0.026). This finding underscores that all laser parameters (power, frequency and cumulative delivered energy) should be cautiously selected and monitored during URS.

This study has several limitations. First, its retrospective design inherently introduces potential biases, limits retrieval of some information such as details on irrigation settings (pressure, manual pump, volume, rate, etc.) and does not warrant a follow-up longer than provided for by institutional guidelines. Second, the laser system does not provide detailed information regarding the mean frequency applied during the operation. This lack of granularity restricts our ability for analysis. Additionally, the study is based on data collected from two institutions, each employing different approaches to URS technique. A difference in surgeons’ expertise between the two institutions cannot be formally excluded. Nevertheless, both are university hospitals from the same country with a urologist with > 100 URS experience present in each surgery, suggesting a minimal impact of possible differences in expertise. Another limitation is the relatively small sample size of 158 patients, which, while sufficient for preliminary observations and findings, may lack statistical power needed to detect subtle associations or rare complications. Despite these limitations, we consider the results of the present study to be robust, as all other significant variables that could influence the rate of US (such as age, ASA score, prior URS history, stone localization and burden, rate of impacted stones) were equally distributed between the two groups. The significant correlation between stone burden parameters (maximal stone size and stone volume) further underscores the quality of the data that was available for analysis. Of note, SFR was not based on systematic postoperative CT scan, potentially leading to an overestimation of success rates. The SFR shall be interpreted accordingly. Larger prospective studies are warranted to confirm our findings and establish safety thresholds for laser settings in lithotripsy.

## Conclusions

High-power, high-frequency settings from manufacturer laser presets significantly increase the risk of US formation, independently from impacted ureteral stones, and without improving SFR. Our findings underscore the importance of careful selection of laser settings during TFL lithotripsy, advocating for individualized laser presets with more conservative settings tailored to each patient and clinical scenario. Future research should prioritize prospective studies to evaluate the specific effects of individual laser parameters, such as power, frequency, cumulative delivered energy and laser energy consumption, in order to identify optimal settings, which can maximize efficacy while minimizing the risk of tissue damage. Additionally, the development of dynamic, real-time adjustable presets based on intraoperative feedback (irrigation flow and temperature, targeting of the stone, laser fiber working distance, stone particle size, stone type, stone ablation rate, etc.) represents a promising avenue for improving patient outcomes.

## Electronic supplementary material

Below is the link to the electronic supplementary material.

Supplementary Material 1

## Data Availability

No datasets were generated or analysed during the current study.

## Abbreviations

ASA: American Society of Anesthesiologists
BMI: Body Mass Index
CI: Confidence Interval
CT: Computer Tomography
DJ: Double-J
HU: Hounsfield Units
IP: Individual Presets
MP: Manufacturer Presets
HUG: Hôpitaux Universitaires de Genève
OR: Odds Ratio
SFR: Stone Free Rate
TFL: Thulium Fiber Laser
UAS: Ureteral Access Sheath
US: Ureteral Stricture
URS: Ureteroscopy
USZ: Universitätsspital Zürich
