# Supplementary material for: With great power comes great risk: High ureteral stricture rate after high-power, high-frequency Thulium fiber laser lithotripsy in ureteroscopy
Source: World J Urol. 2025 Apr 18;43(1):232. doi: 10.1007/s00345-025-05553-0 (PMC12008075; doi:10.1007/s00345-025-05553-0)

| **Supplementary Table 1. Details from the 10 patients with postoperative ureteral stricture (US) after TFL lithotripsy** | | | | | | | | | | | | | | | | | |
| --- | --- | --- | --- | --- | --- | --- | --- | --- | --- | --- | --- | --- | --- | --- | --- | --- | --- |
|  | **Presets** | **Age (years)** | **Stone location** | **Stone size (mm / mm^3^)** | **Impacted stone** | **UAS** | **Active lasing time (mm:ss)** | **Total laser energy (kJ)** | **Laser energy consumption (J/mm^3^)** | **Mean power (W)** | **Maximal power setting (W)** | **Lowest frequency setting (Hz)** | **Highest frequency setting (Hz)** | **Pre- / post-op**  **stenting** | **Stricture location** | **US length (cm)** | **Management** |
| **Patient 1** | IP | 23 | Mid ureter and kidney | 12 mm / 2284 | Yes | Yes | 56:06 | 59.0 | 25.8 | 17.5 | 24 | 5 | 38 | Yes / Yes | Mid ureter | 3 | Nephrectomy (loss of renal function) |
| **Patient 2** | MP | 62 | Mid ureter | 6 / 75 | Yes | No | 01:37 | 1.9 | 25.9 | 20.0 | 20 | 200 | 400 | No / Yes | Distal ureter | 1 | Balloon dilation |
| **Patient 3** | MP | 59 | Mid ureter | 6 / 94 | No | No | 00:35 | 0.7 | 7.5 | 20.0 | 20 | 200 | 200 | No / Yes | Distal ureter | 3 | Laparoscopic DaVinci-assisted reimplantation |
| **Patient 4** | MP | 50 | Mid ureter | 12 / 352 | Yes | No | 05:53 | 7.1 | 20.1 | 20.0 | 20 | 200 | 400 | Yes / Yes | Distal ureter | 0.5 | Spontaneous resolution |
| **Patient 5** | MP | 39 | Proximal ureter | 8 / 100 | No | No | 03:45 | 4.5 | 45.0 | 20.0 | 20 | 100 | 200 | No / Yes | Uretero-pelvic junction | 2.5 | Laparoscopic DaVinci-assisted ureteroplasty with buccal mucosal graft |
| **Patient 6** | MP | 63 | Mid ureter | 17 / 2048 | No | Yes | 39:40 | 141.8 | 69.2 | 59.6 | 60 | 200 | 200 | No / Yes | Uretero-pelvic junction | 1.5 | Balloon dilation |
| **Patient 7** | MP | 46 | Mid ureter | 17 / 748 | Yes | No | 12:28 | 55.6 | 74.3 | 74.3 | 75 | 30 | 100 | Yes / Yes | Mid ureter | 2 | Balloon dilation |
| **Patient 8** | MP | 43 | Proximal ureter | 10 / 330 | Yes | Yes | 03:51 | 4.6 | 14.0 | 20.0 | 20 | 100 | 100 | No / Yes | Distal ureter | 5 | Laparoscopic DaVinci-assisted reimplantation |
| **Patient 9** | MP | 20 | Proximal ureter | 13 / 322 | Yes | Yes | 05:28 | 7.8 | 24.1 | 23.6 | 24 | 200 | 400 | No / Yes | Proximal ureter (total obliteration) | 3 | Laparoscopic DaVinci-assisted ureteroplasty with appendix flap |
| **Patient 10** | MP | 75 | Proximal ureter | 20 / 1497 | Yes | Yes | 24:07 | 52.0 | 34.7 | 35.9 | 40 | 200 | 400 | Yes / Yes | Proximal ureter | 2.5 | Laparoscopic DaVinci-assisted ureteroplasty with buccal mucosal graft |

### Surgical procedure

In both institutions, URS was performed either in general or spinal anesthesia according to patients’ choice and after anesthesiologist counselling. Pre-operative antibiotics were administered 30 minutes before surgery according to institutional guidelines (mostly intravenous cefuroxime or sulfamethoxazole-trimethoprim), or during at least 2 days before surgery according to antimicrobial susceptibility testing in case of positive urine cultures. Since all surgeries were performed in a university hospital setting, each patient was treated by either a urologist with >100 URS experience or by a urology resident under the supervision of an experienced urologist. After rigid cystoscopy and DJ-stent extraction (if present), a safety ureteral guidewire was inserted before URS. According to institutional guidelines, at the HUG, semirigid exploration of the ureter was performed in most cases before insertion of an UAS or flexible URS when a ureteral stone was present, whereas flexible URS was used without prior semirigid URS in most cases at the USZ. The use of an UAS as well as pre- and postoperative DJ-stenting was at the surgeon’s discretion in both institutions. At the USZ, a Flex-Xc or Flex-X2S flexible URS (Karl Storz®, Tuttlingen, Germany) was used together with a Flexor® parallel™ 12/14 F UAS (Cook medical®, Bloomington, Indiana, USA). At the HUG, a Flex-Xc flexible URS (Karl Storz®, Tuttlingen, Germany) or a Single Use Flexible Ureteroscope 7,5 or 9.2 F (PUSEN® PU3033 or PU3022, Zhuhai, China) was used together with a ReTrace® 10/12 F UAS (Coloplast® Group, Humlebaek, Denmark). In both groups, the UAS was inserted co-axially over a working guidewire, which then becomes a safety guidewire after removal of the UAS obturator, according to manufacturers’ instructions. The most noticeable difference between both institutions was the use of individual laser presets (IP) at the USZ [15–17], as compared to manufacturer laser presets (MP) at the HUG (Figure 1). This difference in practice was the basis for a comparison between patients treated at the USZ and HUG.

**Supplementary Fig. 1**

Correlation between maximal stone size and calculated stone volume


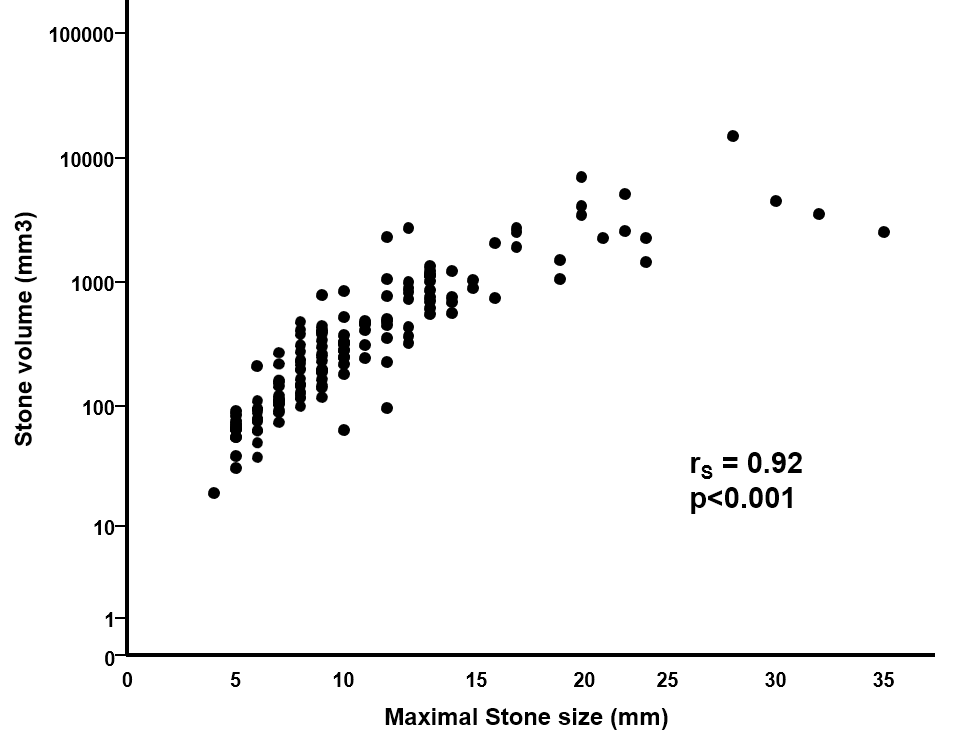

Supplement: Supplementary file 1 — Supplementary Material 1 [file 345_2025_5553_MOESM1_ESM.docx]
